# Characterization of a potential probiotic bacterium *Lactococcus raffinolactis* WiKim0068 isolated from fermented vegetable using genomic and in vitro analyses

**DOI:** 10.1186/s12866-020-01820-9

**Published:** 2020-05-27

**Authors:** Min Young Jung, Changsu Lee, Myung-Ji Seo, Seong Woon Roh, Se Hee Lee

**Affiliations:** 1 Microbiology and Functionality Research Group, World Institute of Kimchi, Gwangju, 61755 Republic of Korea; 2 Division of Applied Life Sciences (BK21 plus), Gyeongsang National University, Jinju, 52828 Republic of Korea; 3 Division of Bioengineering, Incheon National University, Incheon, 22012 Republic of Korea

**Keywords:** *Lactococcus raffinolactis*, Genome sequence, Kimchi, Probiotics, Vitamin B3

## Abstract

**Background:**

*Lactococcus* members belonging to *lactic acid bacteria* are widely used as starter bacteria in the production of fermented dairy products. *From kimchi, a Korean food made of fermented vegetables*, *Lactococcus raffinolactis WiKim0068 was isolated and its genome was analyzed*.

**Results:**

The complete genome of the strain WiKim0068 consists of one chromosome and two plasmids that comprises 2,292,235 bp, with a G + C content of 39.7 mol%. Analysis of orthoANI values among *Lactococcus* genome sequences showed that the strain WiKim0068 has > 67% sequence similarity to other species and subspecies. In addition, it displayed no antibiotic resistance and can metabolize nicotinate and nicotinamide (vitamin B3).

**Conclusion:**

These results augments our understanding of the genus *Lactococcus* and suggest that this new strain has potential industrial applications.

## Background

Lactic acid bacteria (LAB) activity improves the texture, flavor, and scent of dairy products during fermentation and ripening [1]. In these products, LAB starters contribute to flavor development through the (bio) chemical conversion of milk components, such as lactose, fat, casein via glycolysis, lipolysis, and proteolysis, respectively [2]. Moreover, another favorable property of LAB is its ability to adhere to the host intestinal tract which enables them to be effective probiotic strains [3].

The genus *Lactococcus* includes Gram-positive, catalase-negative, non-motile, non-sporulating, cocci-shaped LAB [4]. *Lactococcus* members are widely used as starter bacteria in the production of fermented dairy products, such as cheese and yogurt [5, 6]. Three species within the genus *Lactococcus*—*L. raffinolactis*, *L. lactis* subsp. *lactis*, and *L. lactis* subsp. *cremoris*—are listed among the inventory of microbial food cultures (MFC) of fermented food products as species with demonstrated safety [7].

*L. raffinolactis* is distributed in a wide range of products, including fermented foods such as fish, meat, vegetables, and milk and other materials of plant and animal origin [8, 9]. *L. raffinolactis*, can ferment α-galactosides, such as raffinose and melibios, which are not used by *L. lactis* [10, 11]. The α-galactosides are dominant in soy-derived foods and induce to flatulence and diarrhea. Therefore, fermentation feature of these sugars is a significant advantage for use as a starter in dairy products. In this study, we report the isolation, identification, and characterization of the *L. raffinolactis* WiKim0068 isolated from fermented cabbage (kimchi). We also evaluated the possibility of using the strain WiKim0068 in dairy products, and the safety of the strain. Further, we analyzed its proteolytic enzymes through complete genome sequence analysis. In vitro assays and predictive gene analysis for antibiotic resistance and adhesion were also performed.

## Results

### Phylogenetic and phenotypic features of the isolated LAB strain

**Fig. 1:**
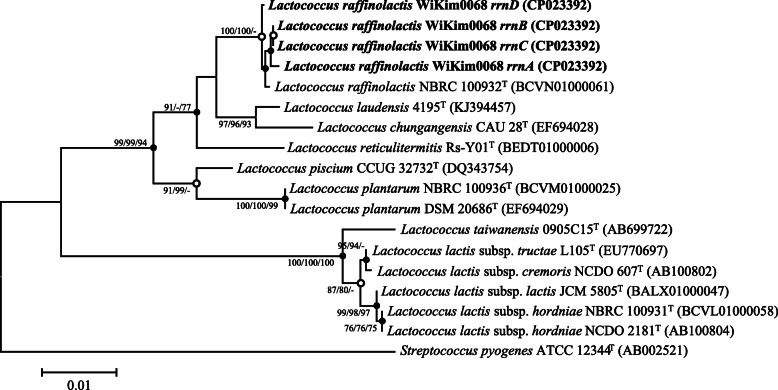
Phylogenetic tree based on 16S rRNA gene sequences showing the taxonomic position of the strain WiKim0068. Numbers at the nodes represent bootstrap values (> 70%) and were calculated using neighbor-joining/minimum-evolution/maximum likelihood probabilities based on 1000 replicates. *Streptococcus pyogenes* ATCC 12344^T^ was used as an out-group. Bar, 0.01 accumulated changes per nucleotides

The bacterial strain, designated WiKim0068, was isolated from a Korean fermented food, kimchi. In order to identify the phylogenetic similarity of the strain, 16S rRNA gene based phylogenetic analysis of strain WiKim0068 was performed and the closely related strains were found to be *L. raffinolactis* NBRC 100932^T^ with a similarity of 99.9% (Fig. 1). This result indicated that strain WiKim0068 belongs to *L. raffinolactis* species. Sugar assimilation/acid formation test conducted using API 50CH revealed positive results for galactose, glucose, fructose, mannose, mannitol, *N*-acetylglucosamine, esculin, ferric citrate, salicin, cellobiose, maltose, melibiose, saccharose, trehalose, raffinose, and turanose, whereas H_2_S production and urease were negative. Enzyme detection performed with an API ZYM kit indicated esterase, leucine arylamidase, and naphthol-AS-BI-phosphohydrolase activities.

### General genomic features of *L. raffinolactis* WiKim0068

The PacBio RS II sequencing system generated 74,558 reads, with an average read length of 8212 bp. The complete genome of the strain WiKim0068 consisted of a circular 2.22 Mb chromosome and two circular plasmids, with a total size of 2.29 Mb. The chromosome contained 2060 predicted protein-coding genes (CDSs), 13 rRNA genes (5S rRNA, 5; 16S rRNA, 4; 23S rRNA, 4), 55 tRNAs, and 3 other RNAs. The WiKim0068 genome was found to contain 39.7 mol% G + C content (Table 1). For functional classification, WiKim0068 genome was analyzed using the clusters of orthologous genes (COG) database (http://www.ncbi.nlm.nih.gov/COG/), and 2000 genes were annotated. The annotated genes were associated with the following categories: general function prediction only (R; 237 genes), carbohydrate transport and metabolism (G; 210 genes), function unknown (S; 190 genes), amino acid transport and metabolism (E; 180 genes), coenzyme transport and metabolism (H; 70 genes), defense mechanisms (V; 63 genes), and secondary metabolites biosynthesis, transport, and catabolism (Q; 15 genes; Supplementary Table S1). In addition, Rapid Annotation using Subsystem Technology (RAST) analysis revealed genes related to the following categories: stress response (2.62%), cofactors, vitamins, prosthetic groups, pigments (5.30%), and virulence, disease, and defense (3.39%) (Supplementary Fig. S1). Stress response-related genes category included: “osmotic stress” (5 genes), “oxidative stress” (17 genes), “cold shock” (1 gene), “heat shock” (15 genes), “detoxification” (9 genes), and “no subcategory” (1 gene). The category of cofactors, vitamins, prosthetic groups, pigments included those related to “biotin” (15 genes), “NAD and NADP” (14 genes), “riboflavin, FMN, FAD” (8 genes), and “folate and pterines” (33 genes). The category of virulence, disease, and defense included mainly those involved in “bacteriocins, ribosomally synthesized antibacterial peptides” (8 genes), “resistance to antibiotics and toxic compounds” (29 genes), and “invasion and intracellular resistance” (15 genes).

**Table 1:** Comparative genomic features of *Lactococcus raffinolactis* WiKim0068, *L. raffinolactis* 4877, and *L. raffinolactis* NBRC 100932^T^

	Strain		
	WiKim0068	4877	NBRC 100932^T^
Assembly size (Mb)	2.29	2.28	2.18
DNA G + C content (mol%)	39.7	38.7	39.8
CDSs*	2187	2362	2123
Scaffolds	3	127	114
Genes	2258	2409	2141
Proteins	2123	2070	2030
rRNAs	13	12	2
tRNAs	55	48	29
Finishing quality	Complete	Scaffold	Contig

*CDSs Coding Sequences

### Comparative genomic analysis

**Fig. 2:**
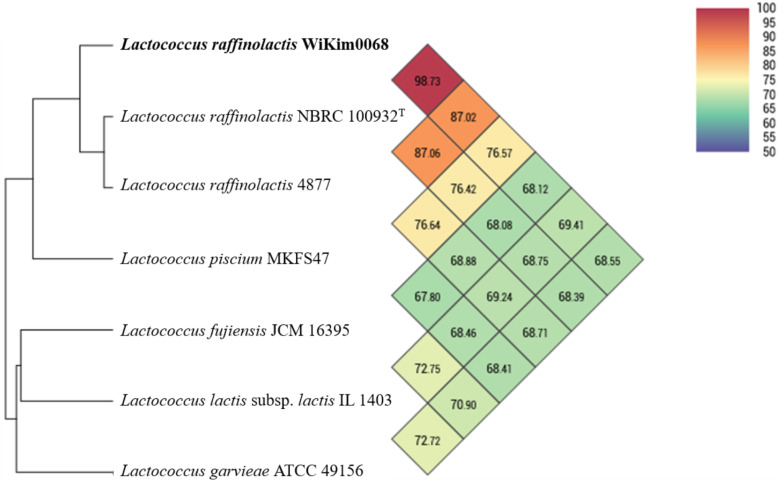
OrthoANI values between *Lactococcus raffinolactis* WiKim0068 and the closely related strains: *L. raffinolactis* NBRC 100932^T^ (98.73%), *L. raffinolactis* 4877 (87.02%), *L. piscium* MKFS47 (76.57%), *L. fujiensis* JCM 16395 (68.12%), *L. lacitis* subsp. *lactis* IL 1403 (69.41%), and *L. garvieae* ATCC 49156 (68.55%). The orthoANI values represent the similarity between the genomes

**Fig. 3:**
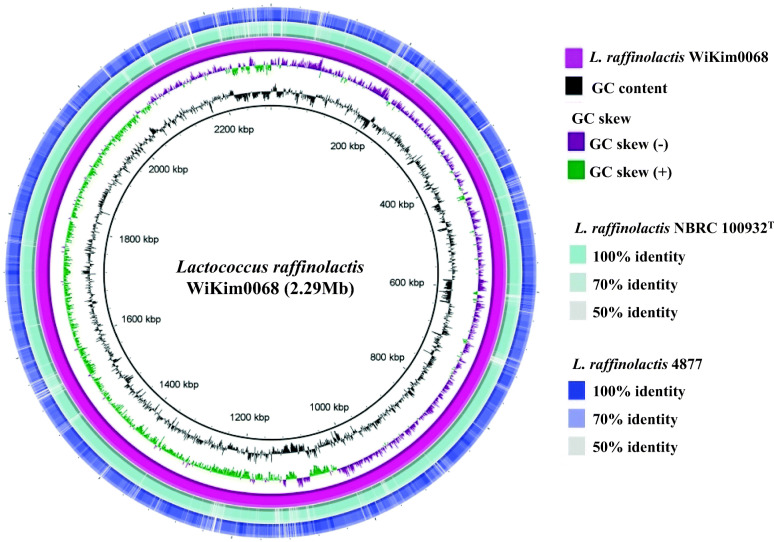
Circular comparison of the genomes of *Lactococcus raffinolactis* WiKim0068 and reference strains, *L. raffinolactis* NBRC 100932^T^ and *L. raffinolactis* 4877. The degree of similarity between the strains is represented by color intensity

Analysis of the orthologous average nucleotide identity (orthoANI) values among *Lactococcus* genome sequences showed that strain WiKim0068 had 68.55–98.73% genome sequence similarities with other species and subspecies. Its genome was most closely related to that of *L. raffinolactis* NBRC 100932^T^ (98.73%), followed by *L. raffinolactis* 4877 (87.02%), *L. piscium* MKFS47 (76.57%), *L. lactis* subsp. *lactis* IL 1403 (69.41%), *L. fujiensis* JCM 16395 (68.12%), and *L. garvieae* ATCC 49156 (68.55%; Fig. 2). Thus, the comparative whole genome sequence analysis indicated that the strain WiKim0068 belongs to the species *L. raffinolactis* [12]. Its similarity to its two closest relative strains (*L. raffinolactis* NBRC 100932^T^ and *L. raffinolactis* 4877), based on BLAST comparison, is shown in Fig. 3. This figure describes the difference in GC contents and similarities between strain WiKim0068 and two closest relative strains. Furthermore, we searched for clustered regularly interspaced short palindromic repeats (CRISPRs) using the CRISPRFinder platform, but no confirmed CRISPRs were found in the WiKim0068 genome.

### Phage and pathogenesis-related genes

PHAST analysis was performed to identify prophage contamination in the genome of WiKim0068. The chromosome contained two intact, one incomplete, and one questionable prophage. The first plasmid (pWiKim0068–1) contained only one incomplete prophage, while the second plasmid (pWiKim0068–2) contained none (Supplementary Fig. S2). Intact prophage regions were located between positions 57,319–90,123 and 1,524,268–1,563,900 bp of the chromosome.

### Metabolic pathway of carbon and amino acid

**Fig. 4:**
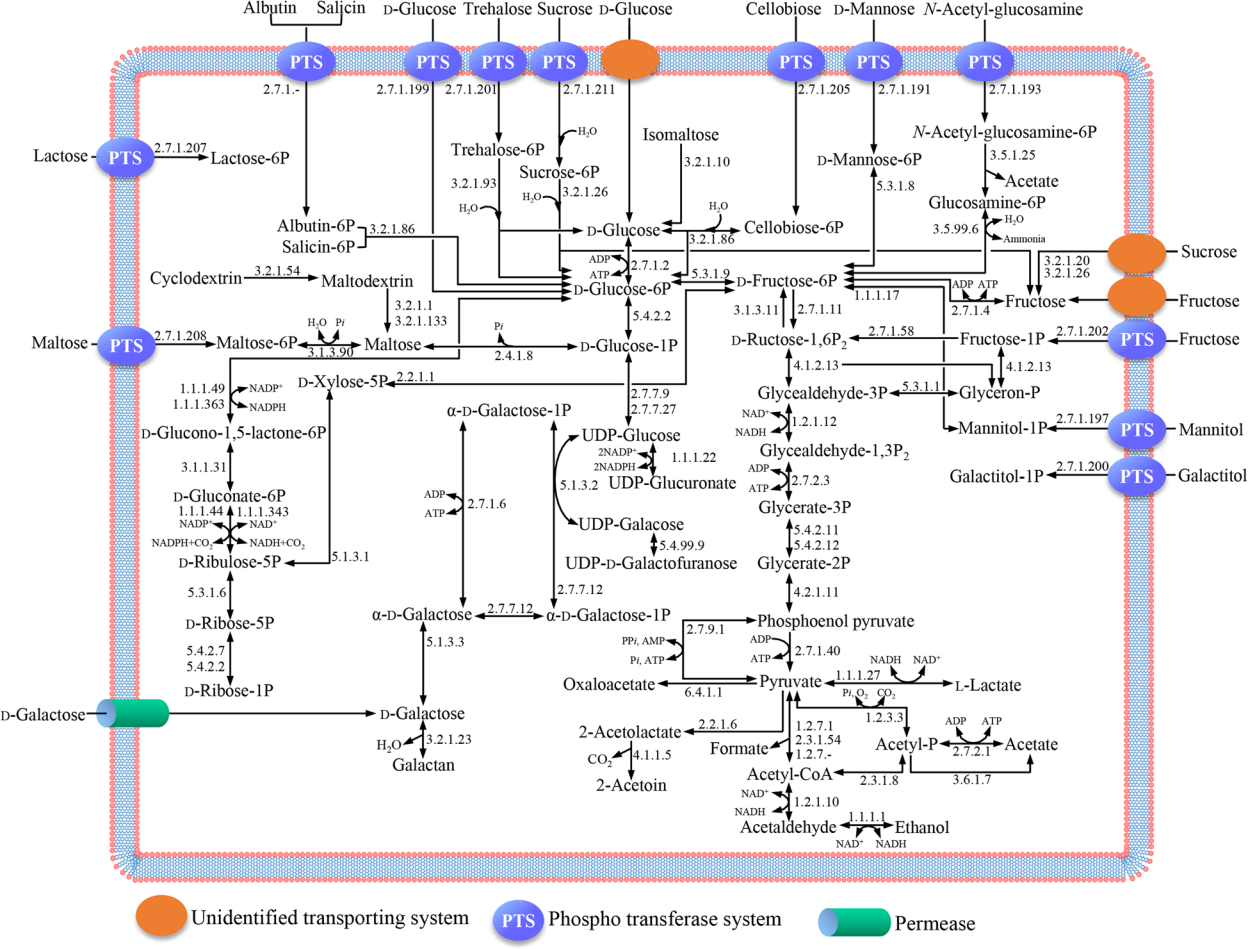
Predicted fermentative metabolic pathways of various carbon compounds in *Lactococcus raffinolactis* WiKim0068 during fermentation. PTS, phosphotransferase systems; UDP, uridine diphosphate

Predicted metabolic pathways in the strain WiKim0068 were associated with diverse phosphotransferase (PTS) systems or permeases that transport various carbohydrates, including d-glucose, d-galactose, d-mannose, trehalose, sucrose, cellobiose, *N*-acetyl-glucosamine, fructose, maltose, mannitol, galactitol, and lactose. The presence of these transport genes suggested that the strain WiKim0068 uses various carbohydrates for fermentation (Fig. 4). Based on the metabolic pathways, it was confirmed that the strain WiKim0068 had heterofermentative pathways.

**Fig. 5:**
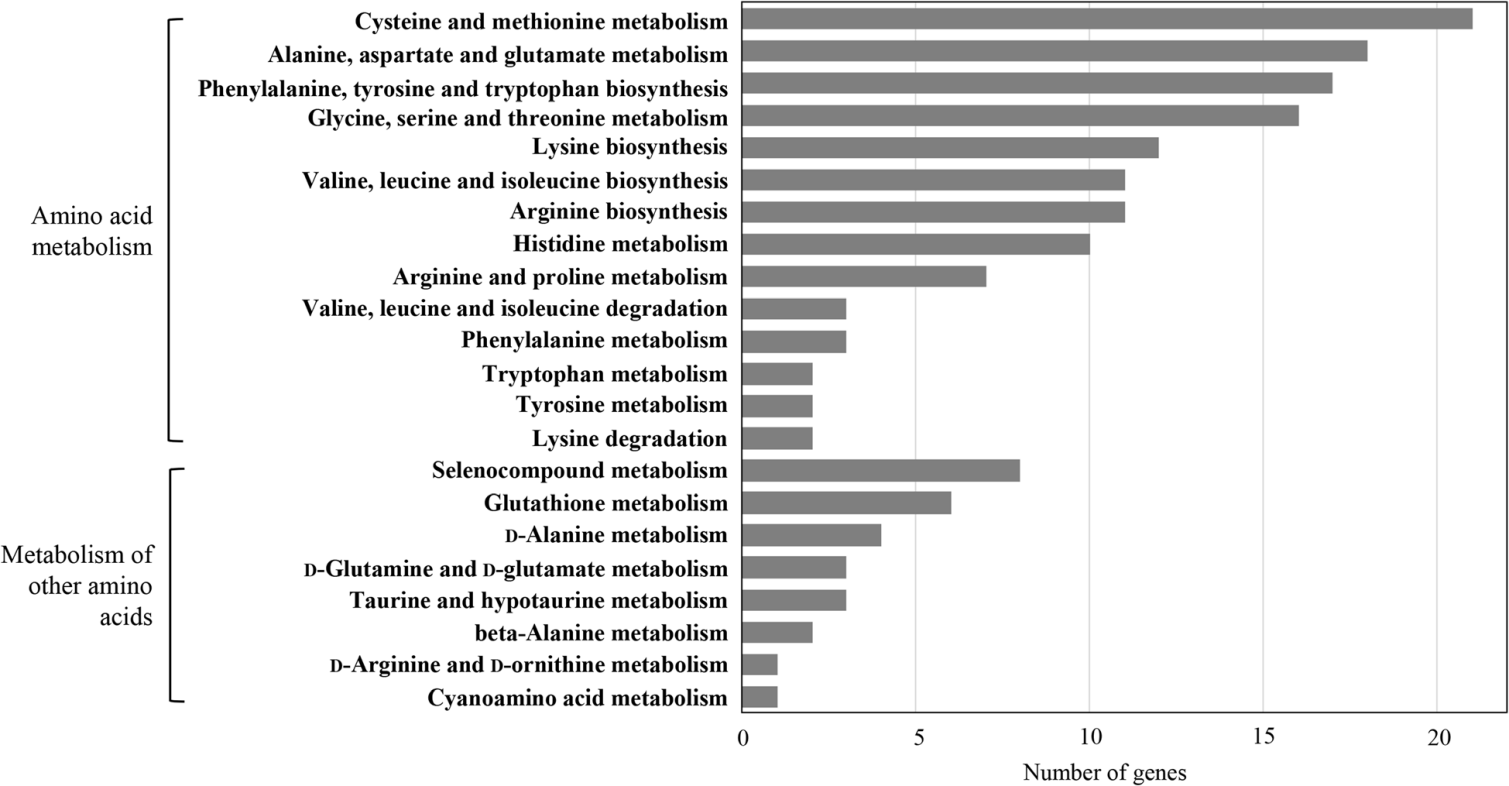
Amino acid metabolism-related genes of *Lactococcus raffinolactis* WiKim0068

The amino acid metabolism-related genes of strain WiKim0068 were annotated using the KEGG database. Among 163 genes involved in amino acid metabolism, strain WiKim0068 harbors the most genes involved in the amino acid metabolism of cysteine, methionine, alanine, aspartate, and glutamate (Fig. 5), suggesting that the strain biosynthesize and utilize various amino acids.

### Metabolism of nicotinate and nicotinamide and antibiotics susceptibility

**Fig. 6:**
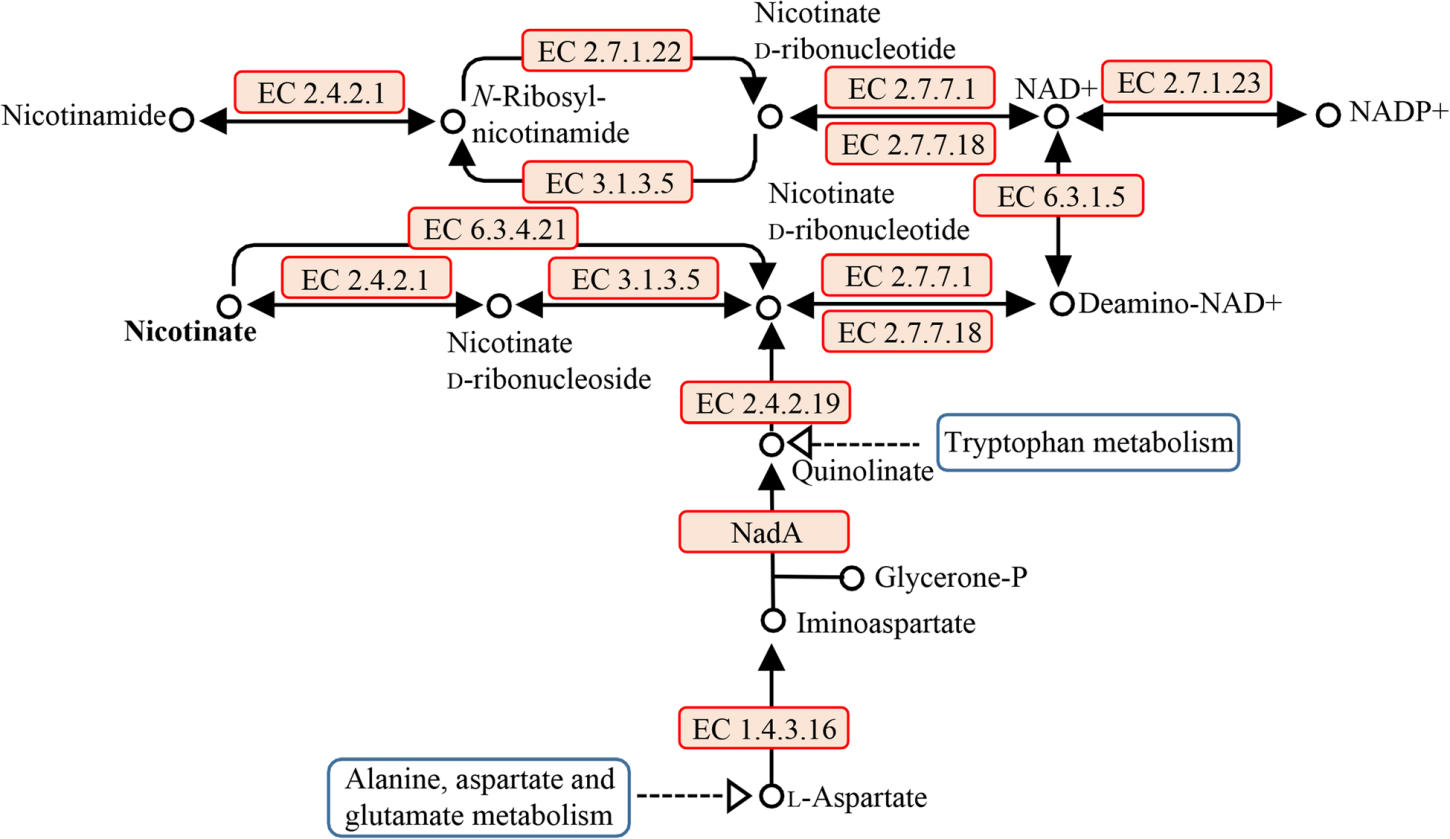
Nicotinate and nicotinamide metabolism in *Lactococcus raffinolactis* WiKim0068. Red boxes indicate enzymes in the nicotinate and nicotinamide metabolic pathway present in the strain WiKim0068

In silico analysis of WiKim0068 genome predicted an almost complete complement metabolic pathway from the genes involved in the metabolism of nicotinate and nicotinamide (Fig. 6). Demonstrating this, 0.932 mg L^− 1^ vitamin B3 was extracted from the cultured cells (Supplementary Fig. S3). These results indicated that nicotinate and nicotinamide metabolism occurs in strain WiKim0068. For comparison, studied 15 LAB isolated from kimchi; *Leuconostoc* spp. produced 0.837–1.05 mg L^− 1^ vitamin B3, and *Lactobacillus* species, *L. sakei*, and *L. curvatus* produced 0.05–0.1 mg L^− 1^. The strain WiKim0068 showed susceptibility to ampicillin, chloramphenicol, ciprofloxacin, erythromycin, gentamicin, penicillin, rifampin, tetracycline, and vancomycin (Table 2).

**Table 2:** Antibiotic susceptibility of *Lactococcus raffinolactis* WiKim0068

Antibiotic	Antibiotic susceptibility test		
	Amount	Inhibition zone diameter (mm)	Resistant/Susceptible
Ampicillin	10 μg	30	Susceptible
Chloramphenicol	30 μg	40	Susceptible
Ciprofloxacin	5 μg	28	Susceptible
Erythromycin	15 μg	48	Susceptible
Gentamicin	10 μg	18	Susceptible
Penicillin	10 U	64	Susceptible
Rifampin	5 μg	42	Susceptible
Tetracycline	30 μg	65	Susceptible
Vancomycin	30 μg	23	Susceptible

## Discussion

The phylogenetic and genomic analysis of strain WiKim0068 confirmed that strain WiKim0068 is closely related to *L. raffinolactis* NBRC 100932^T^ (Fig. 1 and Fig. 2). The G + C content of strain WiKim0068 was 39.7 mol%, which is within the range of 35.5–46.4% reported for *Lactococcus* species [13], and similar to the 39.25 mol% observed in two *L. raffinolactis* strains, 4877 (CALL00000000) and NBRC 100932^T^ (BCVN00000000). In addition, the orthoANI analysis showed that strain WiKim0068 is mostly similar to *L. raffinolactis* NBRC 100932^T^ (98.73%). In the RAST analysis, various genes were identified in the genome of strain WiKim0068. Biotin, riboflavin, and folate are related to human health and digestion and cause various symptoms when deficient [14]. Bacteriocins are antimicrobial peptides produced by bacteria [15] and an alternative to treat antibiotic resistant bacteria. Significantly, bacteriocins production have been regarded as an important feature in the selection of probiotic strains. These were associated with the presence of useful probiotic characteristics, which play important roles in the food and pharmaceutical industries [16–18].

Hexoses (glucose, fructose, and mannose) are converted to lactate, ethanol, and carbon dioxide. Additionally, d- and l-lactate are produced from the reduction of pyruvate by d-lactate dehydrogenase (d-LDH) (EC 1.1.1.28) and l-lactate dehydrogenase (l-LDH) (EC 1.1.1.27), respectively. However, strain WiKim0068 harbors only l-LDH (locus tag: CMV25_RS07125). Notably, as shown in a previous report, l-LDH was identified in *Lactococcus lactis*, which belongs to the same genus as the strain WiKim0068 [19]. Since d-lactate produced by LAB may induce d-lactate acidosis in some individuals [20], it is important to develop LAB for the production of dairy products that produce only l-lactate. Therefore, the lack of d-LDH is an advantage that makes the strain WiKim0068 suitable for potential applications in the dairy industry.

Vitamin B3 production of strain WiKim0068 was identified through in in silico and in vitro analysis. Vitamin B3, one of the 8 B-vitamins, is also known as nicotinate or niacin. This endogenous metabolite is an effective antioxidant that prevents oxidative damage [21]. In general, nicotinamide and nicotinate metabolites are frequently reported in *Lactobacillus* strains [22–24], while *Lactococcus* members were not known to produce these metabolites until now. On the other hand, the capacity to adhere to mucosal surfaces is a useful assay to determine whether probiotic strains have beneficial health effects [25]. Strain WiKim0068 was bound to Caco-2 cell cultures, and its adhesion did not significantly differ from that of *L. rhamnosus* GG (Welch’s t-test, *P* > 0.05) (data not shown). The extracellular proteins of lactobacilli play important roles mediating interactions with the host or the environment [26]. Cell surface proteins of strain WiKim0068 include glyceraldehyde-3-phosphate dehydrogenase, triosephosphate isomerase, trehalose and maltose hydrolases (possible phosphorylases), beta-galactosidase, lipoprotein signal peptidase, and sortase (surface protein transpeptidase), which have been implicated in adhesion or binding to other cells [27].

Recently, interest in foods as mediators of antibiotics resistance has been increasing. LAB, which are widely used in probiotics and as starter cultures, have the potential to serve as hosts for antibiotic resistance genes, and present the risk of transferring genes from various LAB and bacterial pathogens [28]. Although the strain WiKim0068 was predicted to have vancomycin resistant gene in the genome, antibiotics test confirmed that it was sensitive to vancomycin. This result based on the antibiotic resistance gene prediction can be obtained from a cluster of vancomycin resistant genes. Strain WiKim0068 had only *vanW* gene among the vancomycin resistance gene cluster, and the function of this gene is still unknown. The safety of *L. lactis* strains has not yet been assured through the comparison of antibiotic susceptibility profiles and the presence of the genes putatively encoding antibiotic resistance-related proteins [29, 30]. The analysis of *L. raffinolactis* WiKim0068 based on ResFinder 3.0 did not detect antimicrobial resistance genes against aminoglycoside, beta-lactam, colistin, fluoroquinolone, fosfomycin, fusidic acid, glycopeptide, macrolide-lincosamide-streptogramin B, nitroimidazole, oxazolidinone, phenicol, rifampicin, sulphonamide, tetracycline, or trimethoprim. The safety against antibiotic resistance of *L. raffinolactis* WiKim0068 could be confirmed by the antibiotic susceptibility test and antibiotic resistance gene prediction.

## Conclusions

The complete genome of *L. raffinolactis* WiKim0068 revealed its general genomic features, carbon metabolic pathway, and its ability to produce and utilize nicotinate and nicotinamide. In addition, in vitro analysis indicated that the strain possesses beneficial health effects such as vitamin B3 production. These results suggest that *L. raffinolactis* WiKim0068 could be utilized in comparative genome analysis with other *Lactococcus* strains.

## Methods

### Isolation and characterization of the bacterial strain

The strain WiKim0068 was isolated from kimchi, a Korean fermented food, in Gwangju, Korea using the dilution plating method, and incubated on De Man, Rogosa and Sharpe (MRS) agar (MB cell, LA, USA) at 30 °C for 48 h under anaerobic conditions (BD GasPak™ EZ Anaerobe Container Systems, New Jersey, USA). Physiological characteristics (acid production, carbon-source utilization, enzyme activity, and biochemical feature) were determined using the API 50CH, API ZYM, and API 20E galleries (bioMérieux, France), according to the manufacturer’s instructions [31, 32], while the bacteria were incubated at 30 °C for 48 h under anaerobic conditions. Anaerobic conditions were maintained using mineral oil.

### Genome sequencing and annotation

Genomic DNA extraction was performed using the QIAcube system with a QIAamp DNeasy Blood & Tissue Kit (Qiagen, Hilden, Germany). The genome was sequenced using the PacBio RS II sequencing system (Pacific Biosciences, Menlo Park, CA). The reads were assembled de novo using Hierarchical Genome Assembly Process version 3.0 (HGAP 3.0) in PacBio SMRT analysis version 2.3.0., as described by Jang et al. [33]. The complete genome sequence was annotated using the combined results of the automatic National Center for Biotechnology Information (NCBI) Prokaryotic Genomes Annotation Pipeline 4.1 [34] and the RAST server [35]. Phylogenetic tree based on 16S rRNA gene sequences extracted from the genome, were constructed, as described by Ismaeil et al. [36], using the neighbor-joining [37], minimum-evolution [38], and maximum likelihood [39] methods, based on 1000 randomly generated trees. Protein functions were grouped according to COG using WebMGA on-line tools (for carbohydrate metabolism, antibiotic resistance-related genes, adhesion, proteolytic enzymes, and amino acid metabolism) [40]. Nicotinate and nicotinamide metabolic pathway was mapped using the Kyoto Encyclopedia of Genes and Genomes (KEGG) [41]. The fermentative metabolic pathways were constructed based on predicted KEGG pathways and BLASTP analysis using reference gene sequences. Antimicrobial resistance genes were identified using ResFinder 3.0, available from the Center for Genomic Epidemiology (http://genomicepidemiology.org/). Prophage identification was performed using the PHAge Search Tool (PHAST) [42]. The complete genome sequences have been deposited to the DNA databank of Japan/the European Molecular Biology Laboratory/GenBank under the accession numbers CP023392–CP023394.

### Carbon metabolic pathway

The fermentative metabolic pathways of *L. raffinolactis* WiKim0068 were constructed based on predicted KEGG pathways and BLASTP analysis. In detail, the genes of *L. raffinolactis* WiKim0068 were mapped to the five KEGG pathways (pentose phosphate pathway, fructose and mannose metabolism, pathways for pyruvate, galactose, starch, and sucrose metabolism). Then, only mapped genes were used to draw one pathway (Fig. 4), and the functions of the individual genes were reconfirmed using BLASTP.

### Comparative genomic analysis

For comparative genomic analysis of strain WiKim0068, the genome sequences of two other *Lactococcus raffinolactis* strains: *L. raffinolactis* 4877 (CALL00000000.1) and *L. raffinolactis* NBRC 100932^T^ (BCVN00000000.1) were obtained from GenBank and used as references. To determine the similarity between genome sequences, OrthoANI values of *L. raffinolactis* WiKim0068 and related strains in the genus *Lactococcus* were calculated using the orthologous average nucleotide identity tool (OAT software, www.ezbiocloud.net/sw/oat; ChunLab) [43]. Circular comparison map of the genomic sequences was created using Blast Ring Image Generator (BRIG) software [44]. Clustered regularly interspaced short palindromic repeats (CRISPRs) were analyzed using CRISPRFinder [45]. When the algorithm was detected exactly three identical (repeated and sequential) repeating regions separated by a variable order, it was considered “confirmed CRISPR”.

### In vitro analyses

#### Antibiotic susceptibility test

Antibiotic susceptibility was determined by the agar disk diffusion method on MRS agar according to the Clinical and Laboratory Standards Institute (CLSI) guidelines [46]. The 100 μL inoculum (10^7^–10^8^ CFI mL^− 1^) was spread on MRS plates. Antibiotic disks (Becton Dickinson Microbiology Systems, USA) were placed on MRS agar plates, incubated at 30 °C for 48 h, and the diameter of each clear zone was measured in millimeters. Disks containing ampicillin (10 μg), chloramphenicol (30 μg), ciprofloxacin (5 μg), erythromycin (15 μg), gentamicin (10 μg), penicillin (10 U), rifampin (5 μg), tetracycline (30 μg), and vancomycin (30 μg) were used.

#### Adhesion assay

Human colorectal adenocarcinoma cell line Caco-2 (HTB-37) was obtained from the Korea Collection for Type Culture (KCTC). Caco-2 cells were grown in minimum essential medium (MEM) according to KCTC guidelines. Adhesion of bacteria to Caco-2 cells was tested as previously described [47]. Briefly, the strains were added to confluent cell layers (10^6^ CFU well^− 1^) in antibiotic-free cell media. After 2 h of incubation, the cell layer was washed to remove non-adherent bacteria and lysed by the addition of 0.1% Triton X-100 (Sigma-Aldrich, St. Louis, MO, USA). The viable adhered bacteria were plated on LAB Petrifilm (3 M Company, St. Paul, MN, USA) and the cell number was counted after incubation at 30 °C for 48 h. Adhesion experiments were performed in triplicate and *Lactobacillus rhamnosus* GG (KCTC 5033) was used as a control. Statistical evaluation was performed using GraphPad Prism 6.0 (GraphPad Software Inc., La Jolla, CA, USA). Differences were considered statistically significant when *P* < 0.05.

#### Quantitative vitamin B analysis

The strain WiKim0068 was cultured at 30 °C for 48 h in MRS broth under anaerobic conditions. The cell-free supernatant was collected using a 0.22 μm syringe filter. Two microliters of the cell-free supernatant was injected into the HPLC system. Vitamin B levels were determined with a NexeraX2 HPLC (Shimadzu, Japan) equipped with an LCMS-2020 LC/MS System (Shimadzu). The compounds were separated on an Aegispak C-8 column (150 mm × 2 mm, 3 μm; Young Jin Biochrom, Korea) at 40 °C. Mobile phase A was a 0.1% formic acid in distilled water and mobile phase B was 0.1% formic acid in acetonitrile. The gradient elution was as follows: from 0 to 1 min isocratic elution with 100% of mobile phase A, then the mobile phase B content was increased linearly to 75% in 20 min. Finally, the isocratic elution (25% A and 75% B) was continued for 7 min. Solvents were delivered at a total flow rate of 0.25 mL min^− 1^. The re-equilibration time was 5 min. Optimal operating conditions for LC-MS/MS analysis were applied according to the method reported by Wirkus et al. [48]. Reference vitamin B group standards with 99% purity supplied by the Sigma-Aldrich were used. All experiments were repeated at least three times. Statistical analysis was performed using Tukey’s honest significant difference (HSD) test carried out in the “agricolae” package of the R program for group comparisons. Results with *p* < 0.05 were regarded as statistically significant.

## Supplementary information

**Additional file 1: Figure S1.** The subsystem category distribution of strain *Lactococcus raffinolactis* WiKim0068. A total of 1,565 proteins were categorized within these subsystems. **Figure S2.** Prophages of *Lactococcus raffinolactis* WiKim0068 identified using the PHAse Search Tool (PHAST). Intact prophage, red; questionable prophage, green; incomplete prophage, gray. **Figure S3.** Vitamin B concentration in *Lactococcus raffinolactis* WiKim0068. B1, vitamin B1 (thiamin); B6, vitamin B6 (pyridoxine); B3, vitamin B3 (nicotinate); B5, vitamin B5 (pantothenic acid); B12, vitamin B12 (cobalamin); B2, vitamin B2 (riboflavin); B7, vitamin B7 (biotin); B9, vitamin B9 (folic acid). All experiments were repeated at least three times. **Table S1.** Genes associated with general COG functional categories in genome of *Lactococcus raffinolactis* WiKim0068.

## Data Availability

The complete genome sequences have been deposited to the DNA databank of Japan/the European Molecular Biology Laboratory/GenBank under the accession numbers CP023392–CP023394.

## Abbreviations

CDS: Coding sequences
RAST: Rapid Annotation using Subsystem Technology
PHAST: PHAge Search Tool
KEGG: Kyoto Encyclopedia of Genes and Genomes
orthoANI: orthologous Average Nucleotide Identity
COG: Clusters of Orthologous Genes
