# Supplementary material for: Characterization of a potential probiotic bacterium Lactococcus raffinolactis WiKim0068 isolated from fermented vegetable using genomic and in vitro analyses
Source: BMC Microbiol. 2020 May 27;20:136. doi: 10.1186/s12866-020-01820-9 (PMC7251713; doi:10.1186/s12866-020-01820-9)
Supplement: Supplementary file 1 — Additional file 1: Figure S1. The subsystem category distribution of strain Lactococcus raffinolactis WiKim0068. A total of 1,565 proteins were categorized within these subsystems. Figure S2. Prophages of Lactococcus raffinolactis WiKim0068 identified using the PHAse Search Tool (PHAST). Intact prophage, red; questionable prophage, green; incomplete prophage, gray. Figure S3. Vitamin B concentration in Lactococcus raffinolactis WiKim0068. B1, vitamin B1 (thiamin); B6, vitamin B6 (pyridoxine); B3, vitamin B3 (nicotinate); B5, vitamin B5 (pantothenic acid); B12, vitamin B12 (cobalamin); B2, vitamin B2 (riboflavin); B7, vitamin B7 (biotin); B9, vitamin B9 (folic acid). All experiments were repeated at least three times. Table S1. Genes associated with general COG functional categories in genome of Lactococcus raffinolactis WiKim0068. [file 12866_2020_1820_MOESM1_ESM.docx]

**Supplementary Figures and Tables**

- 1. **Supplementary Figures**


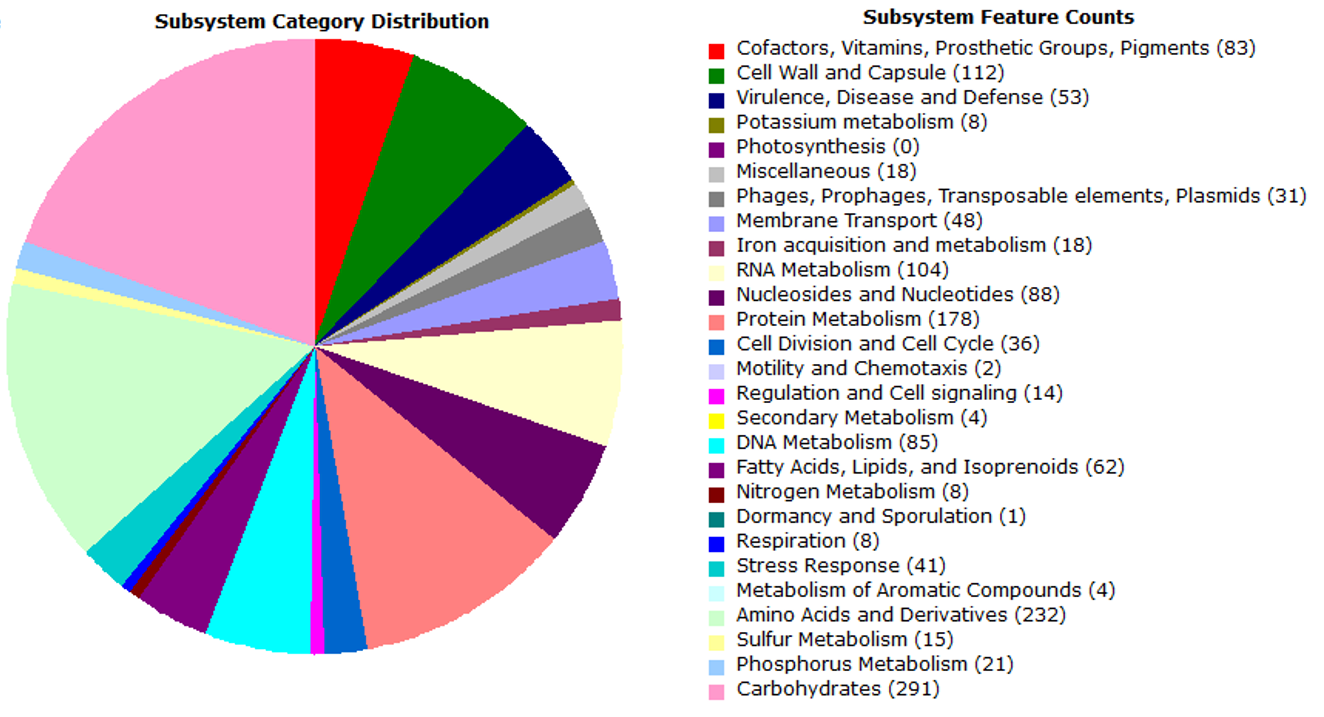


**Supplementary Fig. S1.** The subsystem category distribution of strain *Lactococcus raffinolactis* WiKim0068. A total of 1,565 proteins were categorized within these subsystems.


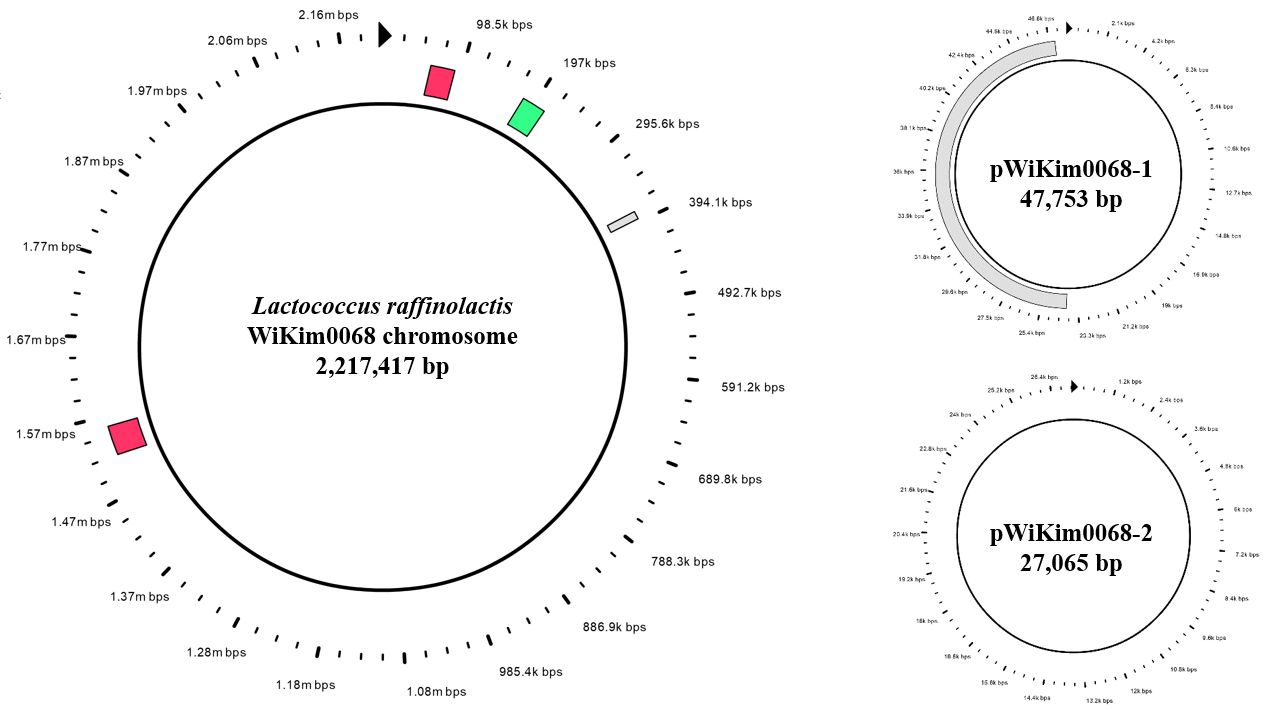


**Supplementary Fig. S2.** Prophages of *Lactococcus raffinolactis* WiKim0068 identified using the PHAse Search Tool (PHAST). Intact prophage, red; questionable prophage, green; incomplete prophage, gray.

**Supplementary Fig. S3.** Vitamin B concentration in *Lactococcus raffinolactis* WiKim0068. B1, vitamin B1 (thiamin); B6, vitamin B6 (pyridoxine); B3, vitamin B3 (nicotinate); B5, vitamin B5 (pantothenic acid); B12, vitamin B12 (cobalamin); B2, vitamin B2 (riboflavin); B7, vitamin B7 (biotin); B9, vitamin B9 (folic acid). All experiments were repeated at least three times.

- 1. **Supplementary Tables**

**Supplementary Table S1.** Genes associated with general COG functional categories in genome of *Lactococcus raffinolactis* WiKim0068.

| **COG** | **Description** | **Number of genes** | **%** |
| --- | --- | --- | --- |
| C | Energy production and conversion | 73 | 3.6 |
| D | Cell cycle control, cell division, chromosome partitioning | 28 | 1.4 |
| E | Amino acid transport and metabolism | 180 | 9 |
| F | Nucleotide transport and metabolism | 80 | 4 |
| G | Carbohydrate transport and metabolism | 210 | 10.5 |
| H | Coenzyme transport and metabolism | 70 | 3.5 |
| I | Lipid transport and metabolism | 42 | 2.1 |
| J | Translation, ribosomal structure, and biogenesis | 146 | 7.3 |
| K | Transcription | 152 | 7.6 |
| L | Replication, recombination, and repair | 158 | 7.9 |
| M | Cell wall/membrane/envelope biogenesis | 108 | 5.4 |
| N | Cell motility | 8 | 0.4 |
| O | Posttranslational modification, protein turnover, chaperones | 54 | 2.7 |
| P | Inorganic ion transport and metabolism | 99 | 4.9 |
| Q | Secondary metabolites biosynthesis, transport, and catabolism | 15 | 0.7 |
| R | General function prediction only | 237 | 11.9 |
| S | Function unknown | 190 | 9.5 |
| T | Signal transduction mechanisms | 61 | 3.1 |
| U | Intracellular trafficking, secretion, and vesicular transport | 26 | 1.3 |
| V | Defense mechanisms | 63 | 3.2 |
| Total |  | 2000 | 100 |
